# The experiences and perceptions of people with chronic and rare diseases during political-economic sanctions in Iran: a qualitative study

**DOI:** 10.1186/s12913-024-10786-7

**Published:** 2024-03-05

**Authors:** Mohammad Mehdi Kiani, Hakimeh Mostafavi, Fatemeh Ebrahimi, Reza Majdzadeh, Efat Mohamadi, Alexander Kraemer, Alireza Olyaeemanesh, Amirhossein Takian

**Affiliations:** 1 Health Equity Research Center (HERC), Tehran University of Medical Sciences (TUMS), Tehran, Iran; 2 Department of Health Management, Policy & Economics, School of Public Health, Tehran University of Medical Sciences (TUMS), Tehran, Iran; 3 Department of Population Medicine and Health Services Research, School of Public Health, Bielefeld University, Bielefeld, Germany; 4 School of Health and Social Care, University of Essex, London, UK; 5 Knowledge Utilization Research Center and Community-Based Participatory-Research Center, Tehran University of Medical Sciences, Tehran, Iran; 6 National Institute for Health Research, Tehran University of Medical Sciences (TUMS), Tehran, Iran; 7 Department of Global Health & Public Policy, School of Public Health, Tehran University of Medical Sciences (TUMS), Poorsina Ave., Tehran, Iran

**Keywords:** Sanction, Health, Chronic diseases, Rare diseases, Health impact, Iran

## Abstract

**Background:**

Economic sanctions aim to exert pressure on political and economic foundations. Hypothesizing that sanctions might affect various aspects of population health, this study, as a component of a broader investigation to ascertain the trend effects of sanctions on selected health outcomes in Iran, seeks to explore the experiences of Iranian citizens associated with the imposed sanctions.

**Methods:**

This is a qualitative study. We conducted 31 semi-structured interviews with randomly selected patients diagnosed with at least one chronic and rare disease from diverse backgrounds across four provinces in Iran. We analyzed data using an inductive content analysis approach, facilitated by the MAXQDA10 software.

**Results:**

We identified three primary themes: direct effects, side effects, and coping strategies. The immediate effects were perceived to be manifested through the restriction of healthcare service availability and affordability for citizens. The side effects included the economic hardships experienced in individuals’ lives and the perceived devastation caused by these difficulties. Some coping mechanisms adopted by patients or their families/relatives included prioritizing comorbidities, prioritizing health needs within families with multiple ill members, and readjusting health/illness requirements in light of daily living needs.

**Conclusion:**

In addition to the inherent burden of their illness, patients faced substantial healthcare costs as a result of sanctions, restricted access to medications, and availability of low-quality medications. We advocate considering these challenges within the healthcare system resilience framework as a crucial first step for policymakers, aiming to determine actionable measures and mitigate the adverse effects of sanctions on citizens, particularly the most vulnerable groups.

**Supplementary Information:**

The online version contains supplementary material available at 10.1186/s12913-024-10786-7.

## Background

Improved nutrition, upgraded and modern medical equipment, and enhanced public health infrastructure are just a few examples of how economic growth can influence public health. Political or economic sanctions have been associated with negative effects on population health indicators. These effects include declining currency value, increasing inflation, and decreasing availability of essential medical supplies, equipment and services [1]. Sanctions have the potential to jeopardize health progress worldwide. For instance, in the former Yugoslavia, the imposition of sanctions resulted in a 10% increase in the country’s overall mortality rate and a 30% increase in hospital mortality due to the collapse of the health system [2].

Since 1979, Iran has been facing numerous political and economic sanctions imposed by various countries [3], which increased severely between 2012 and 2015 [4]. Since May 2018, the United States has unilaterally enforced sanctions to halt Iran’s oil revenue [5]. Furthermore, the European Union’s general restrictions have significantly impeded trade between Iran and its partners across various sectors [6]. Evidence indicates the devastating impact of sanctions on diverse economic aspects, including reduced public revenues from oil exports [7], a decrease in the gross domestic product (GDP), and an increase in the consumer price index [8], and inflation rate [9, 10]. Specifically, the imposed restrictions on Iran’s banking system have reduced the procurement of raw materials for pharmaceuticals and vital high-tech equipment [11].

The Ministry of Health and Medical Education (MOHME) serves as the primary policy-making body, while the universities of medical sciences (UMSs) function as local and provincial authorities responsible for education, research, medicine supply, and the provision of healthcare services to the general public. Official statistics presents a total of 511,238 registered patients with rare diseases in Iran [12, 13]. Furthermore, non-communicable diseases (NCDs) and chronic illnesses accounted for 78.1% of the overall disease burden and 83.5% of all deaths in 2019, indicating the significant burden of these conditions among Iranians [14]. Previous studies have demonstrated that sanctions have had adverse effects on various aspects of public health in Iran, resulting in reduced availability and access to life-saving medications [15–19].

Global evidence shows the impacts of sanctions on lowering life expectancy [20], for instance, an average episode of United Nations’ (UN) sanctions reduces life expectancy by about 1.2–1.4 years [21].

In this regard, various studies have investigated the impact of sanctions on population health [22–24]. Studies in Iran have also shown that patients struggling with rare and chronic diseases such as thalassemia and hemophilia [25], cancer [26], and kidney problems [27] are among the main victims of economic sanctions. From 2012 to 2013, there were significant shortages of medicines to treat diseases such as cancer, heart disease, thalassemia, HIV/AIDS, hemophilia, and Multiple Sclerosis (MS) [28]. Evidence demonstrates a significant deterioration of arthropathy in hemophiliac patients and a significant increase in serum ferritin levels in thalassemia patients in the country [25].

In conjunction with quantitative measurements, additional objective insights are required to provide a comprehensive understanding of the impact of economic sanctions on the Iranian population. Our literature review revealed limited information regarding the experiences and viewpoints of patients residing in countries subjected to political and economic sanctions. To address this gap, our multidisciplinary research team, operating across international borders, conducted a nationwide study to assess the quantitative and qualitative effects of Iran’s economic sanctions on health outcomes over the previous two decades. This paper presents our qualitative findings focusing on the perspectives and experiences of individuals in Iran with rare and chronic illnesses, influenced by sanctions. We intend that the insights gained through this research will contribute to a heightened global awareness of the hardships endured by Iranian civilians, while also having potential relevance in other similar contexts. Moreover, our evidence-based policy recommendations aim to aid in developing tailored health and social protection programs, specifically targeted at assisting the most vulnerable citizens impacted by these sanctions.

## Methods

### Study design and setting

This is a qualitative study. We conducted 31 in-depth semi-structured interviews to explore the perceptions and experiences of patients with chronic and rare diseases during summer 2021 in Iran.

### Data collection and sampling

We developed the generic interview guide (Appendix 1) in repeated discussions within the research team, consisting of all authors and adopted it after two pilot interviews. The interview guide was designed to capture three main directions: [1] the experiences about the effects of sanctions to control and manage the illness conditions, [2] perceptions about the influence of sanctions on the illness condition, and [3] and strategies to deal and cope with the effects of sanctions on the illness conditions.

We deliberately identified 31 patients (18 females, 13 males; aged 20–68) who were registered with the Iranian Health Insurance Organization (IHIO) and diagnosed with one chronic and/or rare disease across four provinces. In order to consider economic and health indicators, we divided the provinces of the country into four clusters. With this in mind, we selected four provinces, namely Tehran (the most affluent), Sistan and Baluchistan (the least affluent), Hamedan, and Yazd (middle), as representatives of each level of development [29]. Since our data collection took place during the peak of the COVID-19 pandemic in Iran (summer 2021), we obtained the participants’ phone numbers from the IHIO, contacted them via phone, and explained the study objectives. We informed them of their right to refuse or withdraw from the study at any time, without any reason. We assured participants about data confidentiality, obtained their verbal consent, and scheduled a mutually convenient time for a telephone interview. The interviews were conducted by three researchers (MMK, HM, FE), lasted approximately 30 min on average, were digitally recorded, transcribed verbatim, and continued until data saturation was achieved. The study population comprised four individuals previously diagnosed with Hemophilia, seven individuals with Diabetes, seven individuals with Thalassemia, nine individuals with a kidney transplant, and four individuals with cancer (see Appendix 2). The exclusion criteria included individuals with communication problems, speech impairments, or cognitive issues.

### Data analysis

We used an inductive content analysis approach [25], comprising several steps including review of materials, creation of notes and headings, and grouping of data. Additionally, we reduced the number of categories by combining similar headings into broader categories. The researcher needed to immerse themselves in the data and identify significant statements throughout this process. Two authors (HM & MMK) independently and concurrently coded the data. Subsequently, the codes were frequently re-read in presence of senior authors until consensus was achieved. We utilized the MAXQDA10 software (VERBI Software, 2020) to store, classify, and manage the data.

## Results

Our analysis identified three themes, seven sub-themes, and 17 codes (see Table 1). Three core overarching themes were: the direct effects of sanctions on health issues, the side effects of sanctions on non-health issues, and the coping strategies.

**Table 1:** The themes, sub-themes and codes

Themes	Sub-themes	Codes
The direct effects of sanctions on health issues	Less affordability of healthcare services	• Ffluctuateing and sharp increase of healthcare services prices • Increasing out-of-pocket payment for healthcare services • Delay in reimbursement by health insurance
	Limited availability and accessibility to medicine	• Difficulties in access to medicines • Shortage of medicines
	Low quality of medicines	• Replacing the high-quality medical products with lower quality • Procurement of medicine from the black market with unreliable quality
The side effects of sanctions on non-health issues	Perceived devastation and Decreased social trust	• Frustration • Instability • Future uncertainty • Distrust and disappointment (distrust to govrnment)
	Life’s economic hardships	• Lower capacity to pay for essential living needs (including meal, medical expenditures, and other commodities)
Coping strategies in response to sanctions’ effects	Re-prioritizing individual and family needs	• Prioritising ones co-morbidities • Prioritising health needs of several sick persons in a family • Re-prioritising living needs vs. health/illness-management needs
	Procrastination	• Ignoring illness symptoms • Delaying illness treatment and management

### Theme 1: direct effects of sanctions on health issues

We identified three impact areas as sub-themes, including reduced affordability of health care services, limited availability and accessibility of health care services, and unsatisfactory quality of health care services. Most participants felt that the cost, availability, and quality of medications and care have often deteriorated dramatically during recent years.

#### Less affordability of healthcare services

Our interviewees noted the increasing cost of some healthcare services such as medications during recent scalation years of sanctions. The reasons include the fluctuating and rapidly rising prices of health care services, increasing co-payments for healthcare services, and delayed reimbursement by health insurance companies. Participants felt frustrated and disappointed with the situation where they could not afford treatment for themselves or their loved ones:

The cost of doctor’s visits has increased. The worst part is that the insurance only covers a small percentage of the costs. The insurance is worthless. Some time ago, my sister had a blood test, out of its 250 tomans cost, the insurance only paid 50 tomans. (Thalassemia patient/Tehran)

Most participants described their concerns and dilemmas in this regard and pointed out to some examples, in which they could not afford the cost of treatment. As a result, many patients had no choice than postponing the treatment plan, or when unavoidable, borrowing from others or selling their properties or other belongings to pay their treatment costs, albeit mostly out of their pocket:

One of my friends needed a prosthesis because of a broken leg. Finally, with the help of relatives and charities, he could have the operation, and, of course, he had to sell something. Unfortunately, if you become sick in Iran, you must mourn for your life and your money. That means you are under all kinds of pressure. (Thalassemia patient/Tehran/26-year-old man)

Our interviewees also highlighted some solutions for coping with financial difficulties in accessing healthcare services, including reprioritizing individual and family needs and procrastination.

#### Limited availability and accessibility to medicines

Economic sanctions negatively impacted the availability and access to medicines for almost all participants, who described difficulties accessing medication shortages.

For the past two years, not only has access to medication become more and more difficult, but they also give us less medication; for example, there used to be five packs, now there are two packs. (Transplant Patient/Yazd)

In the past, a greater number of pharmacies provided medication to patients suffering from rare diseases. However, due to a shortage in the supply of medicines, this service has become restricted to few selected pharmacies. Consequently, patients are now burdened with the inconvenience of undertaking long journeys to access these designated pharmacies, where they are further subjected to extended waiting periods to obtain the necessary medication.

I used to be able to just go to a pharmacy near my home and get my medication, but now I have to travel a long distance to get my medication at the only pharmacy that carries it. I mean, our medications are no longer easily available at all pharmacies. (Hemophilia patient/Hamedan)

#### Low quality of medicines

Most participants were dissatisfied with the quality of medicines and all health services requiring technologies. Due to the shortage of medicines, they had to resort to local products, which were reportedly perceived of poorer quality and had a lower curative effect. Furthermore, for this reason, participants sometimes tried to obtain drugs from the black market, which were more expensive and of unreliable quality.

I think the medications used to be more effective. Now I feel they don’t work as well as they used to. I heard that the original medicines don’t come into the country because of the embargo, and the medicines they give us are similar to them, but they are not as effective as them. (Thalassemia patient/Tehran)

### Theme 2: the side effects of sanctions on non-health issues

As we learned from interviews, sanctions can also affect patients indirectly through the cost of living and the psychological burden of the population.

#### Economic hardships

Due to the economic sanctions and the resulting severe inflation over time, the price of food and other living necessities have become unaffordable for many citizens:

In general, living has become more difficult. Everything has become expensive, making it difficult for us to buy fruit and meat or eat the very least of them. (Hemophilia patient/Tehran)

It has become too expensive to buy meat, fruit, and chicken. We have no way to have fun and to travel, both because of the Corona and the high prices. (Diabetic/Yazd)

In general, we have a hard life. Everything is expensive. If you buy a kilo of fruit, you have to pay 30,000 Tomans. We don’t eat at all or very little meat. (Hemophilia patient/Tehran)

Some participants attempted to meet their basic needs—such as nutrition—by cutting back on other expenses like clothing, entertainment, and travel to offset the living rising costs. Additionally, they put their basic needs ahead of those for managing and treating their diseases.

The prices of goods have put a lot of pressure on us. I only buy clothes once a year because I have other, more important expenses. Chicken and meat have also become very expensive. (27-year-old man Hemophilia patient/Tehran)

#### Perceived devastation and decreased social trust

Some participants expressed their perception of devastation and frustration arising from future uncertainties. They also expressed an increasing pessimism regarding the socioeconomic situation and a heightened sense of uncertainty about what lies ahead of them.

It is unclear what we are paying for and when the situation will improve. I have no hope at all. (Hemophilia patient/Tehran)

Some participants complained from fading trust in the government’s capacity to address the economic issues, which was exacerbated by external pressure on the country. Some patients pointed to sanction as a cruel action which contradicts the principles of human right.

It is very brutal that a patient who has nothing to do with politics dies because of a wrong decision of governments. (thalassemia patient)

### Theme 3: coping strategies in response to the consequences of sanctions

Re-prioritizing the individual and family life was mentioned as the main strategy that patients and their family members took to deal and cope with drastically devastating economic changes in their livelihoods during the past few years.

#### Re-prioritizing individual and family needs

Participants frequently ranked their illnesses and family members according to the severity of the disease and the priority of treatment, considering the cost of living and healthcare services. They tried to prioritize the most severe symptoms and diseases because family financial resources were insufficient to treat multiple illnesses in one person or, in some cases, several patients in the same family. Sometimes they even sacrificed themselves to save other family members.

I am very sick but I hardly take care of my illnesses. Visiting a doctor and buying medicines are very expensive. That’s why I only go to the doctor when I feel really sick or when my medicines are finished. (Diabetic/Yazd)

Many participants thought that sanction affected all aspects of their lives, particularly on treating their patients.

I myself have migraine, digestive and nervous problems, but I have to take care of my son who has thalassemia, because if his treatment is neglected, his condition will worsen. (the mother of Thalassemia patient, 56-year-old)

#### Procrastination

The high cost of living and healthcare costs left patients with no choice but to postpone their treatment, overlooking their health problems and/or delaying their cure to save money:

Currently, I have many health problems. I have symptoms of many diseases, and these have worried me a lot, but because I have to go to the doctor for my blood pressure and thyroid, I have given up on the symptoms of other diseases. (thalassemia patient/Yazd)

## Discussion

This study represents the qualitative inquiry into the impact of economic sanctions on rare and chronic diseases in Iran. Economic sanctions permeate the medical sector [9], influencing the rates of morbidity and mortality among vulnerable demographics [31–34]. They also impose a considerable cost burden on the healthcare system [30–33]. They also impose a considerable cost burden on the healthcare system [34–37].

Our participants reported both direct and indirect consequences of sanctions on their health conditions and life experiences. Our investigation revealed that individuals suffering from chronic and rare conditions were profoundly affected, grappling with the dual challenges of managing their illnesses and enduring the hardships posed by the prevailing economic climate. Our findings illuminate how reduced affordability and constrained access to healthcare services and mounting economic adversities undermine individuals’ quality of life and coping mechanisms.

Similar to previous studies [1, 38, 39], the direct impact of sanctions on population health is perceived to be primarily through restricting affordability, availability, accessibility, which can lead to deteriorating quality of healthcare services. In Iran, evidence has revealed a significant increase of 50–75% in the price of medication as a result of these sanctions [40]. A study conducted during 2012–2013 in Tehran, when sanctions against Iran were intensified, demonstrated supply insufficiencies for imported asthma medications and domestically-produced alternatives in community pharmacies [27]. Furthermore, economic sanctions against Iran have led to shortages of both patented and generic cancer drugs [26].

Additionally, sanctions have adversely affected the quality of healthcare services and medicines. In numerous cases, entry barriers have hindered the availability of specific medications, thereby resulting in the importation of low-quality drugs and equipment [41, 42], alongside an increase in the prices of other medications. Given that approximately 97% of drugs are domestically produced, with 60% of their raw materials being imported [43], the foreign companies’ refusal to sell crucial raw materials to Iran has further exacerbated this predicament. Furthermore, sanctions have compelled physicians to prescribe materials and equipment of inferior quality [43].

The escalating economic sanctions have imposed significant costs on Iran’s medical needs shortages [18]. This has resulted in the acquisition of medicine with questionable quality from the black market [44]. Not only have sanctions made medication for rare diseases less accessible, but they have also diverted patients from the appropriate course of treatment. In addition to their direct impact on population health, sanctions have unintended consequences on the population’s psychological well-being and cost of living. As time passed, feelings of hopelessness, uncertainty about the future, and disappointment have contributed to people’s discontent, mistrust of the government, and a decline in social trust.

As endorsed by previous research [45], our study demonstrates the effects of sanctions on the declining quality of life and the mental health burden in Iran [45], which was further exacerbated by the COVID-19 crisis. The economic pressure from sanctions has also reduced people’s ability to afford essential necessities, including food, medical expenses, and other commodities. Similar studies have highlighted the limited impact of humanitarian exemptions for food, medicines [46, 47] and shelter [48] in improving access to these resources.

Despite government’s efforts to mitigate the adverse effects of sanctions, particularly for vulnerable populations, the economic hardships induced by sanctions have necessitated people to adopt coping strategies to manage the growing burden of related problems. This study enhances our understanding of the strategies employed by individuals in coping with the daily challenges of managing chronic illnesses amidst the repercussions of economic sanctions. To alleviate the increasing burden on patients’ families, we recommend expanding domestic production capacity for medicines, particularly those used to treat rare diseases, and a more meaningful liaison with the civil society and philanthropic organizations to assist the poor in supplying them with needed medications.

### Rigor and credibility of methodology

We tried to increase the soundness of the research process and the resulting interpretations by selecting our participants (patients) from various socioeconomic provinces to ensure capturing a broader range of opinions. In addition, to enhance the validity of the findings, codes and themes were developed and reviewed by all research team members to obtain cross-validation of findings. All team members were outsiders to the research context; they had no prior contact with the participants or the research field. MMK, HM and FE moderated the Interviews, while all the team were aware of the study context. The interdisciplinary research team consisted of 8 Ph.D researchers in field of health policy and public health. MMK, HM, EM and FE who analyzed the data had no relationship with the study participants before conducting the in-depth interviews. Additionally, all authors actively involved in developing and reviewing codes and themes, thereby facilitating cross-validation of the findings. By combining different perspectives and data sources, we assured that more comprehensive and reliable understanding of the phenomenon was established. Also, we tried to mitigate errors by considering the following points during interviews:

- At the beginning of each interview, the interviewers introduced themselves to the participants (that they are the researchers of the research team from the Tehran University od Medical Science and even the code of research ethics was read to them to reassure them that the interview was only for research purposes).
- We did not ask any private question (participants’ contact information was obtained from the IHIO).
- We conciderd the diversity of participants through selecting patients from various provinces.
- We continued the interviews until reaching data saturation.

Nevertheless, our study had some limitations. We employed a convenience sampling strategy involving a limited number of participants [30] drawn from the list of IHIO clients. Consequently, our findings may not be fully representative of all individuals suffering from chronic and rare diseases in Iran. Another limitation is the inability to stratify the patients’ opinions by gender, primarily due to the small number of patients participating in the interviews.

## Conclusions

Sanctions have had a significant impact on patients and other vulnerable groups, affecting various aspects of their lives and presenting significant obstacles. These individuals not only grapple with their existing illnesses but also face limited access to high-quality medications and unwarranted additional costs. Moreover, the burden of increased out-of-pocket expenses for medical care and medication can force patients to reassess their priorities, potentially compromising other essential aspects of their lives or even necessitating the cessation of certain expenses. Identifying the challenges patients face prompts policymakers to explore potential solutions to bolster the internal capacities and resilience of the Iranian health system, as well as analogous contexts. With these challenges duly recognized, policymakers can devise concrete plans to address them, thereby facilitating improvements in the overall functioning and efficacy of the healthcare system.

### Electronic supplementary material

Below is the link to the electronic supplementary material.

Supplementary Material 1

Supplementary Material 2

## Data Availability

According to the ethical approval for this study we are unable to make the data publicly available because was granted based on the premise that all data and will be confidential and stored in protected hardware with limited access for authors. However, the transcripts of interviews in Persian format are stored. Upon request, data can be accessed by contacting the correspond author. To fully anonymize the interview data, a unique code was assigned to each interview.

## Abbreviations

MOHME: The Ministry of Health and Medical Education
UMSs: universities of medical sciences
UN: United Nations
IHIO: Iranian Health Insurance Organization
GDP: Gross domestic product
MS: Multiple Sclerosis
IHMO: Iranian Health Insurance Organization
