# Supplementary material for: The experiences and perceptions of people with chronic and rare diseases during political-economic sanctions in Iran: a qualitative study
Source: BMC Health Serv Res. 2024 Mar 5;24:276. doi: 10.1186/s12913-024-10786-7 (PMC10913614; doi:10.1186/s12913-024-10786-7)
Supplement: Supplementary file 1 — Supplementary Material 1 [file 12913_2024_10786_MOESM1_ESM.pdf]

(Appendix 1): Interview guide, main questions, probing questions

Age<br>Gender<br>Education status<br>Employment		
	**Main questions**	**Probing questions**
1	How do you assess your illness condition in recent years?	• Have you perceived any change in your health and illness condition in recent years?<br>• Have you perceived any difficulties in managing your illness in recent years?
2	Do you think that economic sanctions have had effects on your illness condition in recent years?	• If yes, how it influenced your illness condition?<br>• If not, what else have you perceived as the main reason of the difficulties you've experienced in managing your disease?
3	Do you think that economic sanctions have influenced the health conditions of your family members?	• If yes, how it influenced their health?<br>• If not, what else do you think has been most important in influencing the health status of your family in recent years?

4	Generally, how do you perceive the impact of international economic on Iranian's health?	• If yes, how it influenced the health of population?<br>• If not, what else do you think has been most important in influencing the population health?
5	Looking back, do you think which imposed sanctions have had stronger negative effects on public health, if at all?<br>• Sanctions at the time of Ahmadinejad's presidency<br>• Current sanctions by US president Trump	• Why do you think so?
