# Supplementary material for: The experiences and perceptions of people with chronic and rare diseases during political-economic sanctions in Iran: a qualitative study
Source: BMC Health Serv Res. 2024 Mar 5;24:276. doi: 10.1186/s12913-024-10786-7 (PMC10913614; doi:10.1186/s12913-024-10786-7)
Supplement: Supplementary file 2 — Supplementary Material 2 [file 12913_2024_10786_MOESM2_ESM.pdf]

(Appendix 2): Patients by disease and provinces

Diseases/ province	Tehran	Hamedan	Yazd	Sistan-baluchestan
**Diabetes**	2	1	3	
**Thalassemia**	3		2	2
**Cancer**	2	1	1	
**Hemophilia**		1	2	1
**Kidney transplant**	3	2	3	1
**Total**	31			
